# Histiocytic Sarcoma Associated with Coombs Negative Acute Hemolytic Anemia: A Rare Presentation

**DOI:** 10.1155/2016/3179147

**Published:** 2016-06-26

**Authors:** Sandeep Batra, Stephen C. Martin, Mehdi Nassiri, Amna Qureshi, Troy A. Markel

**Affiliations:** ^1^Department of Pediatrics, Section of Pediatric Hematology and Oncology, Riley Hospital for Children at Indiana University Health, Indiana University School of Medicine, Indianapolis, IN 46202, USA; ^2^Department of Pathology and Laboratory Medicine, Indiana University School of Medicine, Indianapolis, IN 46202, USA; ^3^Department of Surgery, Section of Pediatric Surgery, Riley Hospital for Children at Indiana University Health, Indiana University School of Medicine, Indianapolis, IN 46202, USA

## Abstract

Histiocytic sarcoma (HS) rarely involves extranodal sites, such as the spleen. We report a unique pediatric case of massive splenomegaly and refractory Coombs negative hemolytic anemia (CNHA) secondary to HS. The CNHA resolved completely after an emergent splenectomy. Next generation sequencing (NGS) revealed novel ASXL1, PTPN11, KIT, and TP53 mutations, unmasking a clonal heterogeneity within the same neoplasm.

## 1. Introduction

Malignant histiocytic disorders such as histiocytic sarcoma (HS) are rare in the pediatric population [1]. HS commonly presents with fever, malaise, weight loss, and abdominal pain, but clinical manifestations are varied. HS can be localized or fulminant and uncommonly involves extranodal sites such as skin, bone marrow, soft tissue, and spleen [2]. Fulminant or disseminated HS is often associated with a poor outcome [2].

HS may occur before or after mature B cell lymphomas, acute lymphoblastic, and hairy cell leukemia, suggesting that a common oncogenic cellular origin may exist in some patients [3, 4]. HS can also be associated with autoimmune lymphoproliferative syndrome [5]. The majority of HS express macrophage or histiocytic markers such as lysozyme, alpha-antitrypsin, CD68 (KP1 and PGM1), CD163, CD11c, and CD14 but typically lack Langerhans cell (CD1a and langerin), follicular dendritic cell (CD21 and CD35), and myeloid cell (CD33, CD13, and myeloperoxidase) markers. CD34, CD3, and CD20 (T and B lymphocyte markers, resp.), melanoma (Melan-A, human melanoma black-45, and tyrosinase), and carcinoma (cytokeratin) markers are typically absent [6]. Rarely lineage infidelity may be present and could lead to misdiagnosis of a lymphoma [7].

We report a case of a 17-year-old who presented with massive splenomegaly and severe Coombs negative hemolytic anemia (CNHA). The diagnosis of histiocytic sarcoma (HS) was established after an emergent splenectomy. To our knowledge, HS associated with CNHA in the pediatric population has not been previously reported.

## 2. Case Report

A 17-year-old African American male presented to the local emergency department with fatigue, abdominal pain, jaundice, and worsening pallor, for 2 weeks. On exam, he was pale and icteric and had tender splenomegaly (spleen was palpable 5-6 cm below costal margin). Labs revealed a hematocrit equal to 17%, serum bilirubin equal to 3 mg/dL, a negative Coombs test (direct and indirect), and a platelet count of 39,000. The peripheral smear showed anisopoikilocytosis, abundant spherocytes, tear drop cells, polychromatophilia, with normal appearing granulocytes, and decreased platelets (Figure 2(a)). A bone marrow aspirate revealed a hypercellular marrow, negative for blasts, dysplasia, or hemophagocytosis. The local hematologist initiated treatment with oral prednisone (2 mg/kg/day) and weekly rituximab (375 mg/m^2^ for 4 total doses) and administered 2 doses of intravenous immunoglobulin (1 gram/kilogram body weight given a week apart), based on the presumptive diagnosis of a Coombs negative Evans Syndrome.

**Figure 1:**
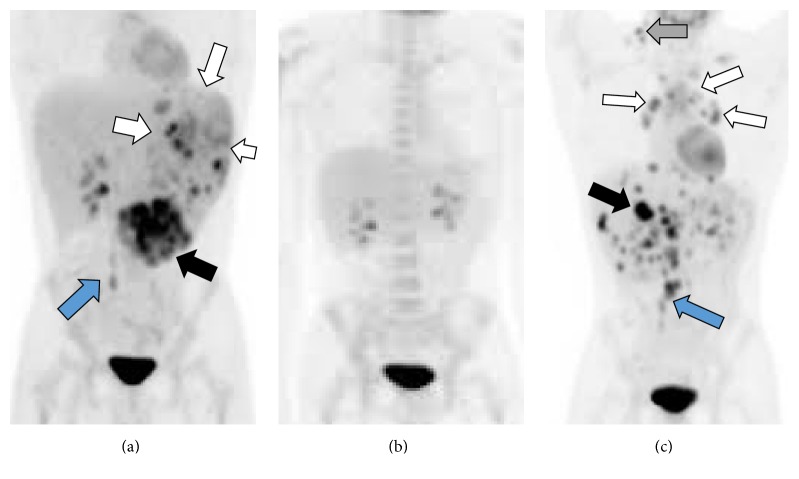
PET scans. (a) Prechemotherapy PET-CT scan (anterior view) demonstrates marked splenomegaly (white arrows), with multiple hypermetabolic foci scattered throughout the spleen and in the retroperitoneum (blue arrow). There was a large aggregate of abnormal foci at the lower edge of the spleen that measured 7 × 6 × 5 cm (black arrow); (b) PET-CT after 6 cycles of CHOP chemotherapy shows no evidence of disease; (c) recurrent hypermetabolic foci and lymphadenopathy involving multiple sites within the liver (black arrow), peritoneum (blue arrow), mediastinum (white arrows), and neck (gray arrow).

However, the hemolytic anemia persisted, and the patient continued to require 2–4 units of packed RBC transfusions to keep the hematocrit >25%. The spleen (now below umbilicus) continued to increase in size and was associated with severe abdominal pain and distension. He was then referred to our hematology service for further evaluation. We obtained additional labs as follows: ALPS panel (negative), cold agglutinins (negative), reticulocyte count (10%), indirect and direct Coombs testing (negative), bilirubin (5 mg/dL, mostly indirect), and serum haptoglobin (undetectable). Flow cytometry on peripheral blood failed to reveal an abnormal blast population and demonstrated a normal RBC expression pattern for CD55 or CD59, excluding acute leukemia or PNH, respectively, as the probable cause of the presentation. The urinalysis was negative for hemosiderin or blood. Infectious work-up for* Bartonella*, brucellosis, tuberculosis, and malaria was negative. We obtained an abdominal PET-CT which demonstrated marked splenomegaly, with multiple areas of heterogeneous and hypermetabolic enhancing foci/masses that were scattered throughout the spleen and also in the retroperitoneum (Figure 1(a)). These findings were suggestive of a lymphoproliferative and neoplastic process.

**Figure 2:**
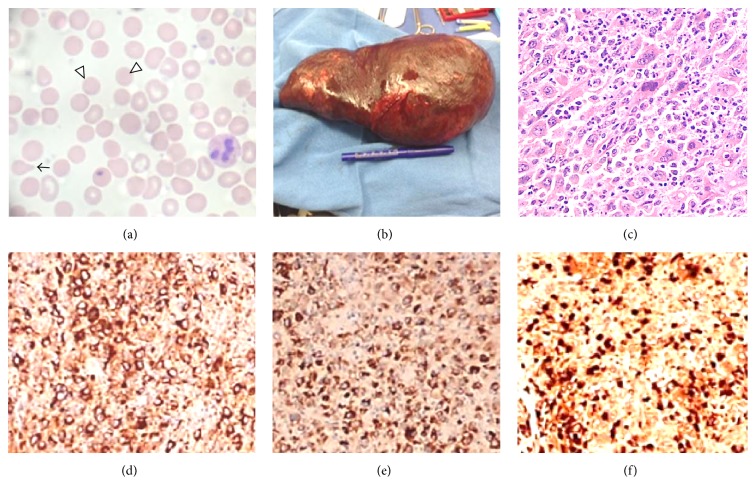
Peripheral blood smear and histopathology of the spleen. (a) Prechemotherapy peripheral blood smear with anisopoikilocytosis, numerous spherocytes (white arrows), tear drop cell (black arrow), and decreased platelets; (b) massively enlarged, surgically removed spleen (weight = 1770 grams; 24 × 14 × 11 cm in size); (c) histological sections of the spleen revealed sheets of atypical cells with a high nuclear to cytoplasmic ratio, prominent nucleoli, and an abundant eosinophilic cytoplasm (400x magnification); (d, e, and f) areas of spleen involved with HS demonstrated strong and diffuse immunoreactivity with macrophage-specific markers (CD68 PGM (d), CD68 KP1 (e), and lysozyme (f)) [7].

An urgent open splenectomy was performed. The surgically removed spleen (Figure 2(b)) was massively enlarged (weight = 1770 grams, 24 × 14 × 11 cm in size) and very firm in consistency. There were several hypertrophied vessels noted in the splenic hilum. Serial sections revealed a red-brown, congested parenchyma, with numerous gray-yellow nodules (0.1–2 cm in size), concentrated at the lower edge of the spleen. A repeat bone marrow aspirate demonstrated a hypercellular marrow, with trilineage maturation and devoid of HS or hemophagocytosis.

Histological sections of the spleen revealed sheets of atypical multinucleated cells with a high nuclear to cytoplasmic ratio, irregular to round nuclear contour, prominent nucleoli, and an abundant eosinophilic cytoplasm (Figure 2(c)), with varying degrees of apoptosis. These neoplastic cells stained positively for CD45, CD68 PGM and Kp1 (Figures 2(d) and 2(e), resp.), CD14, CD23, fascin, and lysozyme (Figure 2(f)) and were negative for CD34, myeloperoxidase, and S100 protein, confirming the diagnosis of HS [7]. CD20, Pax-5, CD3, CD30, keratin cocktail, desmin, factor VIII, factor XIIIa, CD163, CD1a, CD21, CD35, CD123 immunostains, and BRAF^V600E^ mutation were negative.

We also performed hotspot mutation detection by high-throughput next generation sequencing (NGS), using optimized oligonucleotide probes [8]. Specimens were reviewed by a pathologist before processing. Formalin-fixed paraffin-embedded splenic tissue obtained at the time of diagnosis was used to isolate DNA. Fifteen full genes (exons only) as well as additional 39 oncogenic hotspots were analyzed with highly multiplexed next generation sequencing (Illumina TruSight Myeloid Sequencing Panel) (Supplemental Table  1 in Supplementary Material available online at http://dx.doi.org/10.1155/2016/3179147) [8]. Limit of detection of this assay as established by our laboratory is 1-2% mutant alleles. The variants were classified according to previously published guidelines and databases. Low prevalence (<10% of allele frequency) mutations were detected in the ASLX1, KIT, PTPN11, and TP53 genes (Supplemental Table  2), along with multiple variants of unknown significance (not included in supplemental data), suggesting that mutated or variant tumor cells comprised only a minor portion of the malignant clone.

Importantly, the hemolytic anemia and thrombocytopenia resolved rapidly and completely after splenectomy. We then elected to treat our patient with adjuvant CHOP chemotherapy (cyclophosphamide, doxorubicin, vincristine, and oral prednisone) [9, 10]. The PET-CT scans after 3 cycles of CHOP and at the end of therapy (Figure 1(b)) demonstrated no residual or recurrent FDG avid lesions. However, the remission only lasted 3-4 months; recurrent disease was identified in the liver and abdominal lymph nodes (Figure 1(c)) on a PET scan and confirmed with a supraclavicular (neck) lymph node and CT guided liver needle biopsy. Interestingly, the CNHA did not recur with the relapse.

The relapsed disease was treated with 4 cycles of ICE (ifosfamide, carboplatin, and etoposide) chemotherapy [11] followed by an autologous stem cell transplant (using BEAM, carmustine, etoposide, cytarabine, and melphalan, as a conditioning regimen) for consolidation [12]. Unfortunately, our patient remained in remission only for 5-6 months, after transplant, and elected for palliative care.

## 3. Discussion

Acquired Coombs negative hemolytic anemia (CNHA) is rare in the adolescent age group. The differential diagnosis of CNHA (nonimmune hemolysis) associated with splenomegaly, in pediatric patients, includes both inherited and acquired causes such as hemoglobinopathies, RBC enzyme or membrane defects, infections, toxins, HLH, immunodeficiencies, microangiopathies, and uncommonly neoplasms [13, 14]. Splenectomy is rarely performed in these patients but may be indicated in severe refractory immune mediated hemolysis, recurrent splenic sequestration, or hypersplenism [15].

Our patient presented with a Coombs negative hemolytic anemia (CNHA), associated with massive splenic involvement by HS. To our knowledge, this is the first pediatric report describing this atypical presentation. The CNHA could have resulted from the altered circulation, hypoxia, and acidification in the massive spleen. An emergent total splenectomy led to a rapid resolution of CNHA, supporting that notion.

Treatment with rituximab, IVIG, and steroids did not improve the CNHA. This initial lack of response to rituximab is, most likely, due to lack of CD20 expression or the existence of a nonimmune mechanism, such as the extravascular destruction of RBCs in the spleen, or direct cell mediated cytotoxicity [16, 17]. There was no lab evidence of significant microangiopathy (no RBC fragments or schistocytosis) or hemophagocytic lymphohistiocytosis (HLH) (hemophagocytosis, hypertriglyceridemia, low NK activity, or hypofibrinogenemia) [18]. In addition, NGS failed to identify high prevalence (>10%) oncogenic mutations that could explain the pathogenesis or the unique presentation of this HS.

Interestingly, the HS infiltrated spleen harbored mutations in the ASLX1, KIT, PTPN11, and TP53 genes (Supplemental Table  2). Mutations in ASXL1 have been observed frequently in acute myelogenous leukemia and myelodysplastic syndromes and are associated with a worse outcome due to an aberrant hematopoiesis [19]. Genomic profiling of acute myelogenous leukemia has identified somatic variants in both PTPN11 and KIT genes [20] and TP53 mutations [21], implicating resistant pathways that require further investigation [22]. It is plausible that these cooperating mutations contributed to HS relapse, in our patient.

## 4. Conclusion

This case report underscores the importance of ruling out an occult malignancy as a rare cause of refractory CNHA. Splenectomy is an effective option to treat CNHA due to splenic involvement by HS.

## Supplementary Material

The supplemental data lists the genes screened for hotspot mutations using multiplexed next generation sequencing, and the mutations that were detected in the tumor at diagnosis.

## Abbreviations

HS: Histiocytic sarcoma
CNHA: Coombs negative hemolytic anemia
PET-CT: Positron emission tomography-computed tomography
ALPS: Autoimmune lymphoproliferative syndrome
CHOP: Cyclophosphamide, doxorubicin, vincristine, and prednisone
ICE: Ifosfamide, carboplatin, and etoposide
BEAM: Carmustine, etoposide, cytarabine, and melphalan
CD: Cluster of differentiation
BRAF: Serine/threonine-protein kinase B-Raf
HLH: Hemophagocytic lymphohistiocytosis
NGS: Next generation sequencing.
