# Supplementary material for: Histiocytic Sarcoma Associated with Coombs Negative Acute Hemolytic Anemia: A Rare Presentation
Source: Case Rep Oncol Med. 2016 Jun 26;2016:3179147. doi: 10.1155/2016/3179147 (PMC4939191; doi:10.1155/2016/3179147)
Supplement: Supplementary file 1 — The supplemental data lists the genes screened for hotspot mutations using multiplexed next generation sequencing, and the mutations that were detected in the tumor at diagnosis. [file 3179147.f1.docx]

**Supplemental Table 1.**

List of genes screened for hotspot mutations using multiplexed next generation sequencing reaction (Illumina TruSight Myeloid Sequencing Panel). Formalin-fixed paraffin-embedded splenic tissue obtained at the time of diagnosis, was used to isolate DNA.

| ABL1 | CEBPA | HRAS | MYD88 | SF3B1 |
| --- | --- | --- | --- | --- |
| ASXL1 | CSF3R | IDH1 | NOTCH1 | SMC1A |
| ATRX | CUX1 | IDH2 | NPM1 | SMC3 |
| BCOR | DNMT3A | IKZF1 | NRAS | SRSF2 |
| BCORL1 | ETV6/TEL | JAK2 | PDGFRA | STAG2 |
| BRAF | EZH2 | JAK3 | PHF6 | TET2 |
| CALR | FBXW7 | KDM6A | PTEN | TP53 |
| CBL | FLT3 | KIT | PTPN11 | U2AF1 |
| CBLB | GATA1 | KRAS | RAD21 | WT1 |
| CBLC | GATA2 | MLL | RUNX1 | ZRSR2 |
| CDKN2A | GNAS | MPL | SETBP1 |  |

**Supplemental Table 2.**

Results of hotspot mutation detection by high-throughput next generation sequencing on formalin-fixed paraffin-embedded splenic tissue (obtained at the time of diagnosis). Only low prevalence (<10 % of allele frequency) mutations were detected in the ASLX1, KIT, PTPN11, TP53 genes. These findings suggest that tumor cells harboring these mutations, comprised only a minor portion of the malignant clone.

| **Gene** | **Mutation/Variant protein** | **Type** | **Allele frequency (%)** | **Gene function/Variant function** |
| --- | --- | --- | --- | --- |
| ASXL1 | c.1927delG/p.Gly645ValfsTer58 | frameshift | 6.6 | transcriptional repression[1]/unknown |
| KIT | c.1747G>A/p.Glu583Lys[2] | missense | 2.9 | oncogene[3]/unknown |
| PTPN11 | c.188A>C/p.Tyr63Ser | missense | 2.4 | RAS/MAPK regulation[4, 5]/unknown |
| TP53 | 1010G>A/p.Arg337His[6] | missense | 6.1 | Tumor suppressor[7]/unknown |
| PTPN11 | c.1517A>G/p.Gln506Arg | missense | 1.8 | RAS/MAPK regulation[4, 5]/unknown |

**References (supplement):**

1 Sugimoto Y, Muramatsu H, Makishima H, et al. Spectrum of molecular defects in juvenile myelomonocytic leukaemia includes ASXL1 mutations. British journal of haematology. 2010 Jul;150(1):83-7.

2 Fleischman RA. Human piebald trait resulting from a dominant negative mutant allele of the c-kit membrane receptor gene. The Journal of clinical investigation. 1992 Jun;89(6):1713-7.

3 Kirschner MM, Schemionek M, Schubert C, et al. Dissecting Genomic Aberrations in Myeloproliferative Neoplasms by Multiplex-PCR and Next Generation Sequencing. PloS one. 2015;10(4):e0123476.

4 Farrar JE, Schuback HL, Ries RE, et al. Genomic Profiling of Pediatric Acute Myeloid Leukemia Reveals a Changing Mutational Landscape from Disease Diagnosis to Relapse. Cancer research. 2016 Apr 15;76(8):2197-205.

5 Tartaglia M, Niemeyer CM, Fragale A, et al. Somatic mutations in PTPN11 in juvenile myelomonocytic leukemia, myelodysplastic syndromes and acute myeloid leukemia. Nat Genet. 2003 Jun;34(2):148-50.

6 Paskulin DD, Giacomazzi J, Achatz MI, et al. Ancestry of the Brazilian TP53 c.1010G>A (p.Arg337His, R337H) Founder Mutation: Clues from Haplotyping of Short Tandem Repeats on Chromosome 17p. PloS one. 2015;10(11):e0143262.

7 Aubrey BJ, Strasser A, Kelly GL. Tumor-Suppressor Functions of the TP53 Pathway. Cold Spring Harb Perspect Med. 2016;6(5).
